# Effect of a Community Health Worker-Led Intervention on Physical Activity in Adults with Type 2 Diabetes in Primary Health Care in the Brazilian Amazon

**DOI:** 10.3390/ijerph23030276

**Published:** 2026-02-24

**Authors:** Elisa Brosina de Leon, Camila Fabiana Rossi Squarcini, Iasmin Machado Soares, Hércules Lázaro Morais Campos, Rafael Martins da Costa

**Affiliations:** 1 Graduate Program in Human Movement Sciences, Faculty of Physical Education and Physiotherapy, Federal University of Amazonas, Manaus 69067-005, Brazil; iasminmachado1999@gmail.com (I.M.S.); herculeslmc@hotmail.com (H.L.M.C.); rafamc95@yahoo.com.br (R.M.d.C.); 2 Professional Master’s Program in Nursing, Department of Health Sciences, State University of Santa Cruz, Ilhéus 45662-900, Brazil; cfrsquarcini@uesc.br; 3 Interdisciplinary Graduate Program in Rural Studies, Federal University of the Jequitinhonha and Mucuri Valleys, Diamantina 39100-000, Brazil; 4 Institute of Health and Biotechnology, Federal University of Amazonas, Coari 69460-000, Brazil; 5 Department of Physical Education, Health Center, Federal University of Rondônia, Porto Velho 76801-059, Brazil

**Keywords:** community health workers, type 2 diabetes mellitus, physical activity, primary care, health intervention, community-based participatory research

## Abstract

**Highlights:**

**Public health relevance—How does this work relate to a public health issue?**

- Addressing physical inactivity among adults with type 2 diabetes, a major modifiable risk factor for disease progression and mortality.
- Evaluating a scalable, community-based strategy in a low-resource Amazonian context where access to health services is limited.

**Public health significance—Why is this work of significance to public health?**

- Shows Community Health Worker-led interventions can reduce inactivity among adults with type 2 diabetes.
- Supports the Community Health Worker’s key role in diabetes self-management and behavior change in underserved populations.

**Public health implications—What are the key implications or messages for practitioners, policy makers, and/or researchers in public health?**

- Results highlight the need to integrate structured, theory-based CHW interventions into Primary Health Care to improve activity behaviors.
- Findings underscore the need for intersectoral approaches linking health services and urban planning to sustain increases in physical activity.

**Abstract:**

Type 2 diabetes mellitus (T2DM) is a major global public-health problem, and physical inactivity contributes to poor disease control. In settings with limited access to health services, as in the Brazilian Amazon, interventions delivered by Community Health Workers (CHWs) within Primary Health Care (PHC) may offer a pragmatic strategy to increase physical activity (PA). We aimed to evaluate the effect of a CHW-led, theory-based intervention on PA among adults with T2DM in PHC in a cluster-randomized, community-based trial. A total of 274 participants were enrolled (intervention: *n* = 140, control: *n* = 134). CHWs in the intervention group completed a blended training (e.g., asynchronous modules, printed educational materials, and hands-on guidance). They conducted six home visits over six months to support behavior change, including increased PA. PA was measured using the International PA Questionnaire (IPAQ-LF), which assessed active commuting, walking, moderate-to-vigorous PA (MVPA), and total PA. Group-by-time effects were examined using mixed-effects zero-inflated Gamma models. No significant intervention effects were observed for the conditional mean of minutes or the probability of participation in active commuting, walking, or total PA. However, for MVPA, the zero-inflated Gamma model revealed a significant intervention effect on the probability of engaging in activity. The intervention group showed a marked reduction in the likelihood of remaining at zero minutes of MVPA (Odds Ratio = 0.08; 95% CI = 0.01–0.79; *p* = 0.001) compared to the control group, indicating effective behavioral activation among previously inactive participants. These findings suggest that empowering CHWs to deliver structured, theory-driven interventions within PHC can reduce inactivity among high-risk adults with T2DM in underserved communities.

## 1. Introduction

Diabetes mellitus (DM) is recognized as a significant public health problem. In 2021, 6 out of every 100 people worldwide were affected, with 96.0% of these cases being type 2 diabetes mellitus (T2DM) [1]. In middle- and low-income countries, fewer than 40% of people with DM receive treatment, and among those, less than a quarter achieve adequate disease control [2]. In Brazil, although 92.2% of individuals who were aware of their diagnosis were undergoing drug treatment, only 35.8% had adequate control of the disease [3].

The management of type 2 diabetes mellitus in vulnerable contexts remains a significant public health challenge [4]. It is crucial to improve pharmacological and non-pharmacological management of T2DM, including adherence to medication, a balanced diet, and regular physical activity (PA) [5,6,7]. PA is a complex and multidimensional behavior encompassing bodily movements produced by skeletal muscles, which may result in energy expenditure above resting levels [8], yielding benefits such as increased insulin sensitivity, reduced insulin resistance, and lower glycated hemoglobin [9,10,11,12].

People with T2DM consistently show low PA levels [13,14,15], which can be attributed to various functional barriers. These barriers include fatigue, mobility and physical limitations, and other physical impairments, as well as external factors such as adverse weather conditions, time constraints, lack of intention to participate, and insufficient social support [16]. Even when advised, they often show limited behavior change and fail to meet guidelines [17,18], resulting in increased mortality and disability-adjusted life years [11]. This situation underscores the need for proximal, ongoing support from healthcare professionals [19,20].

Primary Health Care (PHC) is key for promoting increased PA with a favorable cost–benefit ratio [21]. Behavior-change interventions in PHC may include lectures, counseling, supervised activities, group exercises, walking programs, or brief in-person or phone advice [21,22]. Despite these strategies, population-level improvements remain limited in vulnerable areas such as the Brazilian Amazon [17,18].

PHC is person-centered, respecting needs and preferences [23]. Community Health Workers (CHWs) strengthen the link between care providers and the community, playing a central role in promoting adoption of healthy behaviors among people with T2DM, including PA, medication adherence, and healthy dietary habits. Their ongoing contact with participants enables personalized guidance and reinforcement over time, facilitating tailored goal-setting that aligns with individuals’ abilities, preferences, and daily realities. By providing culturally appropriate education, practical support, and motivational encouragement, CHWs can help reduce key cognitive and behavioral barriers to initiating physical activity, including low self-efficacy, limited knowledge, and competing life demands. This study aimed to evaluate the effect of a CHW-led intervention on PA and the adoption of healthy habits among T2DM patients in Primary Health Care in the Brazilian Amazon.

## 2. Methods

To address these objectives, we conducted a cluster-randomized, community-based trial as part of a broader project entitled “Community Health Worker–Led Intervention for the Management of Type 2 Diabetes Mellitus in the Interior of Amazonas.” This project was developed by the research group “Estudo da Saúde na Atenção Primária da População Amazônica” (SAPPA) at the Federal University of Amazonas. The study took place between June 2023 and 21 May 2025, and the following sections describe the materials and methods in detail.

The study was conducted in the city of Iranduba, located in the state of Amazonas, approximately 38 km by road from the state capital, Manaus. In 2023, the city had a population of formal workers earning up to 1.9 minimum monthly wages (approximately USD 496.00 per month) and a Human Development Index (HDI) of 0.613 [24].

All procedures were conducted in accordance with ethical standards for research involving human participants, following the Helsinki Declaration and the guidelines of the Brazilian National Health Council, ensuring respect for the principles of autonomy, non-maleficence, beneficence, and justice. Ethical approval was granted by the Research Ethics Committee of the Federal University of Amazonas (approval number 5931.419), and authorization to conduct the study was obtained from the Municipal Health Secretariat of Iranduba. All participants provided written informed consent before enrollment.

### 2.1. Participants

#### 2.1.1. Community Health Workers (CHWs)

Eligible CHWs were those affiliated with PHCs within the urban perimeter of Iranduba, defined as within 200 m of the Municipal Health Secretariat headquarters. All six urban PHCs (with an average cluster size of 45.7 participants per unit, ranging from 33 to 63) agreed to participate and were randomly assigned to either the control group (*n* = 3) or the SAPPA intervention group (*n* = 3).

Since the PHCs were randomized into intervention and control groups using a simple random draw performed by the researchers. All eligible CHWs at each PHC were approached to participate. Recruitment was conducted by the study team to ensure consistent implementation of procedures across intervention and control groups, minimizing potential differential enrolment between arms. In total, 35 CHWs were included in the study, with 17 assigned to the SAPPA intervention group and 18 to the control group. Due to the nature of the cluster design, allocation concealment was not feasible. The inclusion criteria were active CHWs working in the selected PHCs and residing in Iranduba. The exclusion criteria included CHWs without patients diagnosed with T2DM under their care at the start of the training, or those on vacation or leave at that time.

#### 2.1.2. Patients with Type 2 Diabetes Mellitus

Each CHW selected, for convenience, patients diagnosed with T2DM in their area of coverage, based on the lists of registered patients available at their PHC. To minimize potential selection bias arising from CHW-led convenience sampling, several safeguards were implemented. CHWs were instructed to approach all eligible individuals within their catchment areas who met the predefined inclusion criteria, rather than selectively recruiting participants based on convenience or perceived willingness. Eligibility criteria were clearly defined and uniformly applied across all recruitment sites. There were 140 participants in the intervention group and 134 in the control group.

The inclusion criteria were participants aged 18 years or older with a confirmed diagnosis of T2DM for at least 6 months, receiving continuous diabetes medication, and under regular follow-up by a CHW. Exclusion criteria included physical limitations that would prevent engagement in physical activity, as well as communication or cognitive impairments that could hinder understanding of the intervention. Additionally, participants were excluded if they relocated to another city or state during the study period.

### 2.2. Intervention

#### 2.2.1. Theoretical–Practical Training

The training program was structured according to the goal-setting theory for self-management proposed by Locke and Latham [25,26]. It combined asynchronous video lessons with a collaboratively developed printed educational booklet. Together, these materials were designed to provide a coherent and accessible framework for CHWs engaged in diabetes care.

The theoretical component consisted of 6 h of recorded instruction, delivered across 7 asynchronous modules to CHWs via WhatsApp. One additional in-person session complemented the remote content, creating a flexible learning environment that allowed CHWs to revisit the material at their own convenience. Each participant also received a printed booklet containing all topics covered throughout the training.

The modules addressed the following themes:

(a) Type 2 diabetes: importance of glycemic control, medication adherence, healthy eating, and physical activity.
(b) The role of CHWs in managing patients with T2DM.
(c) Encouraging active user participation in T2DM management.
(d) Supported self-care approaches for people living with T2DM.
(e) The importance and implementation of an action plan for T2DM management.
(f) Personal strategies for adopting healthy habits.
(g) Collective strategies for adopting healthy habits.

The practical component consisted of CHWs following up patients over six months to apply the theoretical concepts. Each CHW conducted six scheduled home visits to provide guidance on incorporating healthy habits and to measure blood pressure and body weight. Intervention fidelity was monitored through a structured visit-reporting system. After each home visit, CHWs completed and submitted a standardized visit report documenting the visit and the main components delivered. These reports allowed the research team to monitor adherence to the intervention protocol and verify whether the intended six home visits were conducted.

The central objective of the study was to encourage adherence to physical activity at each participant’s level. The intervention was designed to respect personal capacities, limitations, and baseline physical activity status, aiming to promote gradual and feasible engagement in physical activity rather than a standardized or prescriptive approach. This individualized perspective was fundamental to ensure adherence, safety, and sustainability of the proposed activities.

#### 2.2.2. Data Collection Instruments

Given the multifactorial nature of T2DM and the influence of social and clinical determinants on disease management, the considered variables were:

(a) Sociodemographic data included sex (male/female), age (in years), marital status (with spouse/partner or without spouse/partner), race/skin color (black/ brown or white and others), education (in years of schooling), and employment status (yes or no) [27].
(b) Clinical variables included the duration of T2DM (in months/years), number of continuous medications used, and number of comorbidities.
(c) Anthropometric variables used were waist circumference and Body Mass Index (BMI), measured according to standard procedures by the World Health Organization [28,29].
(d) Behavioral Variables:
  - Physical activity: PA was assessed using the International Physical Activity Questionnaire-Long Form (IPAQ-LF), a validated instrument for monitoring PA across various domains [30]. To evaluate the specific impact of the intervention on voluntary health behaviors, analyses were restricted to the transportation (Section 2) and recreation, sport, and leisure-time (Section 4) domains [30]. Data on occupational and domestic PA were excluded to isolate behaviors amenable to the CHW-led intervention.
  - The four specific outcomes were calculated in minutes per week (min/week) as follows: (1) Active Commuting: Calculated as the sum of minutes spent walking and cycling for transportation purposes (Section 2); (2) Walking: Defined as the time spent walking specifically for recreation, health, or fitness (Section 4, distinct from transport walking); (3) Moderate-to-Vigorous PA (MVPA): Calculated as the sum of minutes spent in moderate-intensity activities (excluding walking) and vigorous-intensity activities within the leisure-time domain (Section 4); (4) Total PA: Derived as the cumulative sum of minutes reported for active commuting, leisure-time walking, and leisure-time MVPA.
  - Dietary habits: using the SISVAN Food Consumption Markers Form, the healthy and unhealthy eating practices based on foods consumed (beans, fruits, vegetables, hamburgers, soda or other sweetened beverages, pasta, and candies) were measure on the previous day, with response options of “yes,” “no,” or “don’t know” [31].
  - Self-management of diabetes: assessed using the Diabetes Self-Care Questionnaire (QAD), translated and validated for the Brazilian population. The instrument evaluates six domains of diabetes self-care: “general diet,” “specific diet,” “physical activity,” “blood glucose monitoring,” “foot care,” “medication use,” and “smoking”, across 15 items. Participants report the frequency of behaviors over the past 7 days using a 0–7 scale, with higher scores indicating better self-care [32].

### 2.3. Procedures

Following approval by the UFAM Ethics Committee and the Iranduba Municipal Health Secretariat, the study was introduced to the PHC Coordinator and PHC managers, and initial meetings with CHWs were held. Patients in the SAPPA intervention group were monitored through periodic visits conducted by CHWs, whereas patients in the control group received only routine care from the PHCs.

Data collection was conducted during home visits to patients with T2DM by CHWs from both groups. The process occurred over four days (two for the control group and two for the SAPPA intervention group). Evaluation questionnaires were administered using KoboToolbox for digital structuring and the ODK mobile application (operating offline on smartphones and tablets) to facilitate data collection. All participants were assessed at baseline and at the six-month follow-up.

**Figure 1:**
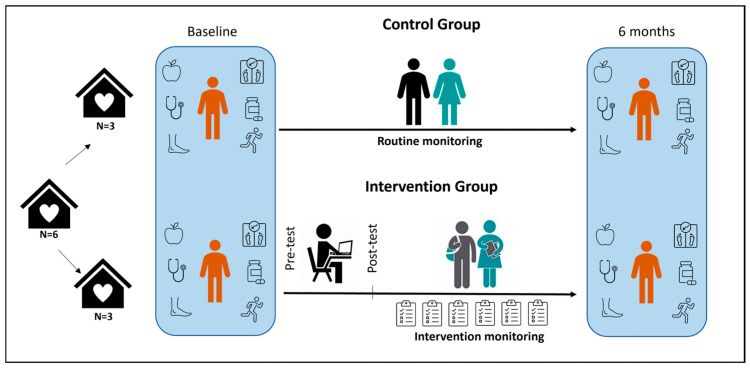
Infographic of the SAPPA Iranduba 2024 Study Development.

An infographic summarizing the six-month training and implementation process is presented in Figure 1.

### 2.4. Statistical Analysis

Participant baseline sociodemographic and clinical characteristics were summarized and stratified by intervention arm (SAPPA intervention group vs. Control group). Continuous variables were presented as mean and standard deviation (SD) or median and Interquartile Range (IQR), depending on the results of normality assessments. Categorical variables were presented as frequencies and percentages. The global primary outcome of the SAPPA trial was diabetes self-management (assessed by the QAD score). The physical activity domains analyzed in the present study (i.e., active commuting, walking, MVPA, and total PA) were pre-specified as secondary endpoints. Therefore, the analyses of these domains are considered exploratory, and no adjustment for multiple comparisons was applied. The intervention effect was evaluated based on the group-by-time interaction term.

Given the longitudinal design with repeated measures (pre- and post), all outcomes were analyzed using Generalized Linear Mixed-Effects Models (GLMMs), which account for the non-independence of observations clustered within individuals. A random intercept for participant ID was specified in all models. To isolate the intervention effect, all models were adjusted for the following a priori-defined potential confounders: sex, age, marital status, skin color, income, duration of T2DM, number of medications, number of comorbidities, fruit and vegetable consumption, and baseline QAD score.

A two-part zero-inflated Gamma “hurdle” model was fit using the glmmTMB package. This approach simultaneously models two processes: (1) a binomial (logistic) component to estimate the odds of a participant reporting any activity versus zero (the “zero-inflation model”), and (2) a Gamma (log-link) component to estimate the mean amount of activity, conditional on the participant reporting a non-zero value (the “conditional model”). Results for the binomial component are presented as Odds Ratios (ORs) with 95% Confidence Intervals (95% CIs), and for the conditional component as Mean Ratios (MRs) with 95% CIs.

To assess the potential influence of the cluster-randomized design, a sensitivity analysis was conducted by adding a random intercept for the PHC unit. However, these models resulted in singular fits with negligible variance estimates at the PHC level; therefore, the final models retained only the participant-level random intercepts to ensure statistical stability.

All statistical analyses were conducted using R statistical software (Version 4.5.2; R Foundation for Statistical Computing, Vienna, Austria). All tests were two-sided, and a *p*-value < 0.05 was considered statistically significant. Given the correlated nature of the physical activity domains and the hypothesis-driven design of the study, no formal adjustments for multiple comparisons were applied; interpretations focused on the magnitude of effect sizes and confidence intervals.

## 3. Results

A total of 274 participants were included in the study, with 140 in the SAPPA intervention group and 134 in the control group. Participants were predominantly female (60.0% in the intervention group vs. 67.2% in the control group, *p* = 0.098), with a mean age of 61.3 ± 11.8 years in the intervention group and 62.4 ± 11.8 years in the control group (*p* = 0.284). Most participants were married or in common-law marriages (56.4% in the intervention group and 62.7% in the control group, *p* = 0.351), and the majority self-identified as Brown/Black (*n* = 223) (Table 1).

Educational attainment differed significantly between groups, with the SAPPA intervention group having lower years of education (mean of 6.8 ± 4.1 years) compared with the control group (8.3 ± 4.4 years) (*p* < 0.001). Employment status was similar between groups, with approximately 28% employed in both (*p* = 0.877). Mean monthly income was 855.4 ± 786.7 BRL in the intervention group and 936.4 ± 813.9 BRL in the control group (*p* = 0.236) (Table 1).

About the clinical characteristics and lifestyle behavior, the participants of the SAPPA intervention group had a shorter duration of T2DM than the control group (8.3 ± 7.3 years vs. 10.0 ± 8.6 years; *p* = 0.012) and used a higher number of medications (2.6 ± 1.9 vs. 2.0 ± 1.5; *p* < 0.001), whereas the number of comorbidities was similar between groups (1.84 ± 1.5 vs. 1.8 ± 1.0; *p* = 0.683). Regular consumption of fruits and vegetables was reported by 62.9% and 77.7% of participants in the intervention group, respectively, and 65.7% and 79.1% in the control group, with no significant differences (*p* = 0.719 and *p* = 0.892, respectively). The self-management of diabetes in the SAPPA intervention group (51.9 ± 13.4) was significantly higher than in the control group (44.4 ± 16.2; *p* < 0.001) (Table 1).

Table 2 presents the unadjusted longitudinal outcomes for both groups at the pre- and post-intervention time points. Descriptive results for the pre-intervention period showed the median active commuting in the intervention SAPPA group was 0.0 min per week (IQR = 0.0–90.0), increasing to 20.0 min per week (IQR = 0.0–102.5) post-intervention, indicating an overall rise in active commuting levels within the group. In the control group, median active commuting remained at 0.0 min per week at both time points (IQR = 0.0–60.0 pre-intervention and 0.0–70.0 post-intervention), indicating minimal change.

Walking time in the intervention SAPPA group increased in the upper quartiles, from a median of 0.0 min per week (IQR: 0.0–22.5) at baseline to 0.0 min (IQR: 0.0–85.0) post-intervention, while the control group showed no change (median 0.0, IQR: 0.0–0.0). Median MVPA remained 0.0 min per week in both groups, reflecting low engagement in higher-intensity activities.

Total physical activity also increased descriptively in the intervention SAPPA group, rising from 27.5 min per week (IQR: 0.0–180.0) at baseline to 115.0 min per week (IQR: 0.0–290.0) after the intervention. In the control group, a minor increase was observed, from 0.0 min per week (IQR: 0.0–116.0) to 30.0 min per week (IQR: 0.0–150.0). These patterns suggest a general upward shift in activity levels in the intervention group, particularly among initially less active individuals, as indicated by the expansion of the upper quartiles.

Beyond the medians, the widening interquartile ranges in the intervention group reflect a tangible increase in the proportion of participants adopting active behaviors. Specifically, the prevalence of participants reporting non-zero Total PA values decreased from 71.4% at baseline to 57.1% post-intervention. Similarly, engagement in Walking decreased from 40.0% to 27.9%, and Active Commuting from 56.4% to 47.9%.

**Figure 2:**
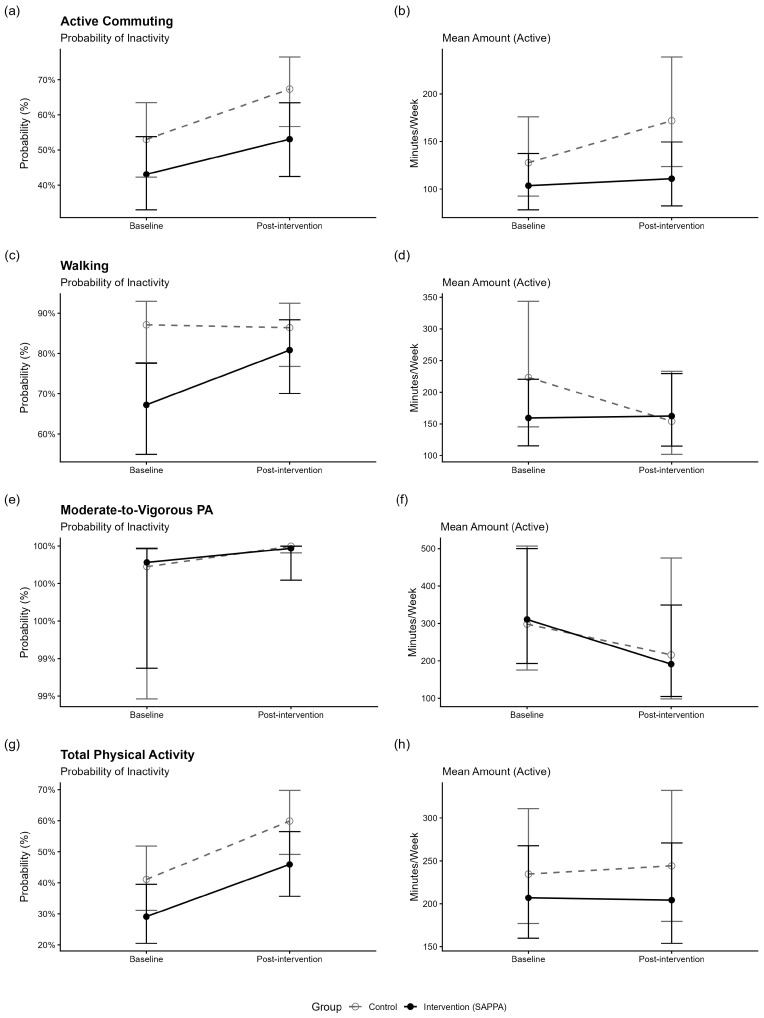
Estimated marginal means of the adjusted mixed-effects models to verify the effect of the SAPPA intervention on physical activity (PA) outcomes in adults with type 2 diabetes mellitus (*n* = 274), Amazonas, Brazil, 2024. (**a**) Zero-inflation model for active commuting; (**b**) Conditional model for active commuting; (**c**) Zero-inflation model for walking; (**d**) Conditional model for walking; (**e**) Zero-inflation model for moderate-to-vigorous PA; (**f**) Conditional model for moderate-to-vigorous PA; (**g**) Zero-inflation model for total PA; (**h**) Conditional model for total PA.

Table 3 presents the results of the adjusted mixed-effects models evaluating the effects of the SAPPA intervention on physical activity outcomes. Figure 2 shows the estimated marginal means from the adjusted models. Analyses using zero-inflated Gamma regression indicated that the time-by-group interaction was not statistically significant for most outcomes, including active commuting, walking, and total physical activity, in both the conditional and zero-inflation components. For MVPA, a significant interaction was observed in the zero-inflation component (*p* = 0.001), with an odds ratio of 0.08 (95% CI = 0.01; 0.79), indicating that participants in the SAPPA intervention group had 92% lower odds of reporting zero minutes compared with changes observed in the control group. In descriptive terms, this effect reflects a substantial mobilization of inactive individuals: From 116 participants with zero min/week of MVPA of SAPPA group at baseline, seven participants transitioned from zero to some MVPA (representing 6.0% of those initially inactive), compared to only four (3.6%) in the control group (from 112 participants in this group with zero min/week of MVPA at baseline).

A sensitivity analysis accounting for PHC-level clustering confirmed the robustness of these findings. The inclusion of a PHC random effect yielded negligible variance estimates (approaching zero), resulting in an Intraclass Correlation Coefficient (ICC) of <0.001, indicating that the intervention effect was independent of specific health unit characteristics.

## 4. Discussion

Analyzing PA patterns among people with T2DM receiving PHC in the Brazilian Amazon, this study demonstrated that a CHW-led intervention significantly influenced PA behavior. Specifically, the adjusted zero-inflated Gamma models identified a significant reduction in the proportion of participants reporting zero minutes of MVPA in the SAPPA intervention group. Given that physical inactivity is a modifiable risk factor associated with the increasing global burden of T2DM [11], this finding is clinically relevant. By empowering CHWs to engage directly with their communities, the intervention successfully initiated participation among individuals who were previously inactive, consistent with literature supporting the benefits of PA for glucose and lipid metabolism, physical fitness, cognitive function, and quality of life [6,9,10,11,12].

Regarding the number of inactive individuals observed in this study, such behavior aligns with the prevalence of physical inactivity among people with DM receiving PHC in the Amazon region [18]. It reflects a broader global trend in which nearly one-third of the population is physically inactive [33]. Notably, physical inactivity tends to be more prevalent among individuals over 60 years of age and women [18,33], both of which were predominant characteristics among the participants in this study.

The strategic positioning of CHW at the interface between the health system and vulnerable communities was a central driver of these results. Systematic reviews indicate that CHW-led interventions improve self-management and health behaviors, particularly in low-income and racially marginalized groups [33,34]. Another advantage of the CHWs’ work lies in its favorable cost–benefit ratio for specific health conditions, especially in low-income communities with limited access to services and predominantly composed of racial and ethnic minorities [34]. In the SAPPA study, the participant profile, mostly Brown/Black with low educational attainment, matches populations where such interventions have demonstrated the most significant impact and cost-effectiveness [34,35]. Furthermore, qualitative evidence suggests that trust-based relationships and shared cultural backgrounds allow CHWs to influence behavior more effectively than sporadic clinical contacts [34,36]. The scheduled home visits provided a platform for longitudinal engagement, allowing CHWs to reinforce goals and adapt recommendations to household realities.

The primary behavioral mechanism underpinning the intervention design was Goal-Setting Theory [25]. The training protocol explicitly instructed CHWs to help participants transform generic health recommendations into concrete, manageable action plans. For a population with lower educational attainment, this structured approach likely reduced the cognitive demands of self-regulation, facilitating the initiation of activity. Beyond this core design element, other theoretical frameworks offer plausible, albeit speculative, interpretations for the observed results. For instance, the transition from zero to non-zero MVPA aligns conceptually with the Transtheoretical Model (TTM) [36], representing a shift from precontemplation to action. Similarly, the supportive, non-judgmental nature of the CHW home visits may have fostered a sense of relatedness and autonomy—key components of Self-Determination Theory [37]. We acknowledge, however, that because specific psychological constructs (e.g., stages of change, self-efficacy, or autonomous motivation) were not measured, these mechanistic explanations remain hypothetical and warrant confirmation in future mechanistic trials.

In the present study, the role of CHWs in navigating the baseline heterogeneity of the target population was evident. The intervention group presented with a sociodemographic profile typically associated with barriers to behavior change, i.e., significantly lower educational attainment (6.8 ± 4.1 years) than the control group (8.3 ± 4.4 years). Yet these participants demonstrated the greatest responsiveness in initiating MVPA. This finding reinforces the “inverse equity hypothesis” often observed in community-based interventions: peer-based support is particularly effective for individuals with lower health literacy who may be underserved by traditional clinical communication [33]. By translating complex management guidelines into accessible, culturally relevant goals, CHWs likely mitigated the disadvantage of lower formal education. Furthermore, although the intervention group had a shorter duration of T2DM, they exhibited significantly higher baseline self-management scores (QAD), suggesting a “gateway effect” [36]. Existing engagement in one domain of self-care (diet/medication) may have provided a behavioral foundation of self-efficacy, upon which CHWs successfully scaffolded new physical activity habits.

Several complementary behavioral mechanisms explain why the intervention effectively moved participants from zero to some MVPA. The training delivered to CHWs was grounded in goal-setting theory [25], which likely reduced the cognitive demands of self-regulation for this population by transforming generic advice into concrete plans. Additionally, the Transtheoretical Model (TTM) [37] provides a framework for interpreting this shift as a progression from precontemplation to action. Repeated visits and personalized counseling likely enhanced task-specific self-efficacy, facilitating the adoption of short bouts of MVPA even amidst comorbidities. Simultaneously, autonomy-supportive counseling promoted the internalization of health goals [38,39]. By encouraging shared decision-making, CHWs supported the basic psychological needs of autonomy, competence, and relatedness, which are essential for behavioral engagement according to Self-Determination Theory [38].

In the present study, the importance of CHWs in selecting community members with T2DM for participation was clearly evident. Consciously or not, CHWs in the SAPPA intervention group tended to recruit individuals with fewer years of formal education, shorter disease duration, and higher medication use than those in the control group, despite these participants demonstrating greater self-management of diabetes. By giving CHWs a leading role in the study, this approach emphasizes recognizing them as essential members of the healthcare team. It highlights their critical contribution to promoting health, preventing disease, and supporting ongoing primary care.

From a clinical perspective, the reduction in participants reporting zero MVPA is significant, even in the absence of large changes in total PA volume. Meta-analyses indicate that moving from inactivity to low or moderate levels of activity yields the most significant relative gains in glycemic control and mortality reduction, reflecting a nonlinear dose–response relationship [10]. Mechanistic studies further support that bouts of exercise improve insulin sensitivity and endothelial function [9,12]. Moreover, in the context of the SAPPA study, this shift likely helped participants reinterpret MVPA not as an externally imposed prescription but as a meaningful strategy to preserve functional independence.

Despite the favorable shift in MVPA, the intervention did not significantly alter active commuting, leisure-time walking, or total PA. This selective effect suggests that different PA domains are constrained by distinct determinants beyond the reach of an individually focused intervention [19,40]. Active commuting and walking are heavily influenced by the built environment, including street connectivity, safety, and infrastructure [41,42]. In the Amazonian setting of this study, structural barriers such as unpaved streets, extreme heat, and long distances are likely to have suppressed walking behavior, consistent with findings from other tropical and Latin American regions. These environmental constraints disproportionately affect low-income groups and underscore the need to complement individual counseling with urban planning and transport policies to achieve broader increases in activity [43,44].

The findings also highlight a critical distinction between initiating behavior and maintaining habits. The intervention appeared sufficient to stimulate sporadic MVPA (as an early action stage change) but may not have provided the duration of support needed to establish robust walking or commuting habits [37,45]. Habit formation often requires months of consistent repetition in supportive environments [45]. In this study, the six-month duration might have been enough to encourage simple, home-based exercises suggested by CHWs, but insufficient to overcome the complex barriers to sustained daily transport-related activity. Furthermore, self-report instruments like the IPAQ-LF may have captured intentional bouts of MVPA more accurately than incidental walking, potentially contributing to the differential findings across domains [46,47].

Analyzing these results through a salutogenic lens reveals that the intervention likely strengthened participants’ sense of coherence regarding MVPA [48,49]. By co-developing culturally adapted materials and centering the intervention on everyday life, CHWs helped participants perceive physical activity as comprehensible, manageable, and meaningful. This approach aligns with evidence that a strong sense of coherence supports health behaviors even under economic hardship. However, while individual and interpersonal resources were enhanced, the lack of community-level “generalized resistance resources”, e.g., such as safe sidewalks and accessible public transport, remained a limiting factor for walking behaviors [49,50].

Taken together, this study demonstrates the potential of assigning CHW a central role in promoting meaningful active habits within the Brazilian context and in the management of T2DM. A key strength of the analytical approach used in this study (i.e., the two-part hurdle model) is its ability to distinguish between two distinct behavioral processes: the decision to initiate activity (crossing the ‘hurdle’ of inactivity) and the degree of engagement among those who are already active. Our findings suggest that the SAPPA intervention successfully helped physically inactive individuals take the first step from ‘doing nothing’ to ‘doing something’ (behavioral activation). However, it did not significantly increase the duration or intensity of exercise among those who were already active. This distinction is clinically vital, as it pinpoints the initiation of movement as the primary mechanism of the intervention’s impact, providing a more nuanced understanding than simple comparisons of mean activity levels.

Nevertheless, this study has limitations that warrant consideration. First, the short follow-up period (six months) precludes conclusions about long-term habit maintenance. Second, the reliance on self-reported measures (IPAQ-LF) introduces potential recall bias that may have differentially affected the outcomes. Self-reports capture structured, intentional activities, such as the MVPA bouts encouraged by CHWs, more accurately than incidental, lower-intensity behaviors, such as habitual walking [45,46]. Consequently, the observed initiation of MVPA might, in part, reflect the greater cognitive salience of these new ‘planned’ activities compared to routine background movement. Third, the recruitment strategy relied on convenience sampling by CHWs, which led to baseline disparities: specifically, the intervention group had lower educational attainment and a lower duration of T2DM. While this selection bias limits the use of strict randomization, it is noteworthy that these characteristics, often markers of therapeutic resistance, did not prevent the intervention from succeeding. In fact, the positive response in this “harder-to-reach” profile suggests that the CHW-led strategy is robust against socioeconomic disadvantages. The predominance of elderly women limits the generalizability of findings to younger men or other demographic groups. Finally, as PA domains were secondary endpoints of the larger trial, these analyses should be interpreted as exploratory. The absence of multiple testing adjustments implies that while the strong statistical signal for MVPA initiation is promising, it requires replication in trials primarily powered for physical activity outcomes.

## 5. Conclusions

This community-based trial conducted in the Brazilian Amazon demonstrates that empowering CHWs to deliver a structured, theory-based intervention was effective specifically in initiating physical activity among previously sedentary individuals (behavioral activation), rather than generating large increases in total activity volume. This finding validates the role of CHWs as essential agents of change capable of helping high-risk, ‘hard-to-reach’ patients with T2DM take the crucial first step away from inactivity. However, the lack of significant gains in active commuting and walking duration suggests that individual counseling alone is insufficient to sustain high levels of activity. Future initiatives must link primary care behavioral activation with broader, intersectoral urban planning strategies to convert this initial momentum into sustained, population-level health improvements.

This finding validates the role of CHWs as essential members of the primary care team capable of reducing health inequities. However, the lack of change in walking and active commuting suggests that broader, intersectoral strategies addressing environmental determinants are required to produce substantial increases in total PA. Future initiatives should link primary care with urban planning to convert initial behavioral activation into sustained, population-level health improvements.

## Figures and Tables

**Table 1:** Sociodemographic and clinical baseline characteristics of adults with type 2 Diabetes Mellitus, SAPPA Study, Amazonas, Brazil, 2024.

Variables	SAPPA Group (*n* = 140)	Control Group (*n* = 134)	*p*-Value
	n (%)	n (%)	
Sex			0.098
Female	84 (60.0)	90 (67.2)	
Male	56 (40.0)	44 (32.8)	
Age (years—mean [SD])	61.3 (11.8)	62.4 (11.8)	0.284
Marital status			0.351
Single/Divorced/Widowed	61 (43.6)	50 (37.3)	
Married/Common-law marriage	79 (56.4)	84 (62.7)	
Skin color			0.269
Brown/Black	118 (84.3)	105 (78.4%)	
White/Indigenous/Yellow	22 (15.7)	29 (21.6)	
Years of education (mean [SD])	6.8 (4.1)	8.3 (4.4)	<0.001
Employed			0.877
Yes	40 (28.6)	37 (27.6)	
No	100 (71.4)	97 (72.4)	
Income (USD *—mean [SD])	855.4 (786.7)	936.4 (813.9)	0.236
Time with T2DM (years—mean [SD])	8.3 (7.3)	10.0 (8.6)	0.012
Number of medications used (units—mean [SD])	2.6 (1.9)	2.0 (1.5)	<0.001
Number of comorbidities (units—mean [SD])	1.84 (1.5)	1.8 (1.0)	0.683
Fruits consumption			0.719
Yes	88 (62.9)	88 (65.7)	
No	52 (37.1)	46 (34.3)	
Vegetables consumption			0.892
Yes	108 (77.7)	106 (79.1)	
No	31 (22.3)	28 (20.9)	
QAD score (19–95—mean [SD])	51.9 (13.4)	44.4 (16.2)	<0.001

*—Value referring to the conversion from Brazilian Real (R$) to US Dollar (USD) on 11 May 2024. SD = Standard deviation.

**Table 2:** Physical activity domain (median and interquartile range—IQR) before and after the study (*n* = 274), Amazonas, Brazil.

Variables	SAPPA Group		Control Group	
	Pre-Intervention Median (IQR)	Post-Intervention Median (IQR)	Pre-Intervention Median (IQR)	Post-Intervention Median (IQR)
Active commuting (min/week)	0.0 (0.0, 90.0)	20.0 (0.0, 102.5)	0.0 (0.0, 60.0)	0.0 (0.0, 70.0)
Walking (min/week)	0.0 (0.0, 22.5)	0.0 (0.0, 85.0)	0.0 (0.0, 0.0)	0.0 (0.0, 0.0)
MVPA (min/week)	0.0 (0.0, 0.0)	0.0 (0.0, 0.0)	0.0 (0.0, 0.0)	0.0 (0.0, 0.0)
Total PA (min/week)	27.5 (0.0, 180.0)	115.0 (0.0, 290.0)	0.0 (0.0, 116.0)	30.0 (0.0, 150.0)

**Table 3:** Effect on physical activity outcomes among adults with type 2 Diabetes Mellitus (*n* = 274), Amazonas, Brazil, 2024.

Outcomes	Time Effect for the SAPPA Group	Time Effect for the Control Group	SAPPA vs. Control Time Effect Contrast	*p*-Value
Active commuting (min/week)				
Conditional model (MR [95%CI])	1.07 (0.48; 2.39)	1.35 (0.94; 1.91)	0.79 (0.51; 1.25)	0.320
Zero-inflation model (OR [95%CI])	1.49 (0.42; 5.29)	1.83 (1.08; 3.11)	0.82 (0.39; 1.70)	0.589
Walking (min/week)				
Conditional model (MR [95%CI])	1.02 (0.33; 3.14)	0.69 (0.42; 1.14)	1.48 (0.79; 2.74)	0.218
Zero-inflation model (OR [95%CI])	2.05 (0.42; 10.05)	0.94 (0.47; 1.87)	2.18 (0.88; 5.39)	0.091
MVPA (min/week)				
Conditional model (MR [95%CI])	0.62 (0.12; 3.20)	0.72 (0.35; 1.51)	0.85 (0.34; 2.12)	0.728
Zero-inflation model (OR [95%CI])	6.99 (0.10; 510.81)	84.52 (11.10; 643.83)	0.08 (0.01; 0.79)	<0.001
Total PA (min/week)				
Conditional model (MR [95%CI])	0.99 (0.47; 2.06)	1.04 (0.76; 1.43)	0.95 (0.63; 1.44)	0.803
Zero-inflation model (OR [95%CI])	2.07 (0.57; 7.40)	2.14 (1.25; 3.63)	0.97 (0.46; 2.04)	0.929

## Data Availability

The data supporting the findings of this study are available upon request from the corresponding author.

## Abbreviations

The following abbreviations are used in this manuscript:

T2DM	Type 2 Diabetes Mellitus
CHWs	Community Health Workers
PHC	Primary Health Care
PA	Physical Activity
IPAQ-LF	International Physical Activity Questionnaire—Long Form
MVPA	Moderate-to-Vigorous Physical Activity
DM	Diabetes mellitus
SAPPA	Estudo da Saúde na Atenção Primária da População Amazônica
HDI	Human Development Index
BMI	Body Mass Index
QAD	Diabetes Self-Care Questionnaire
SD	Standard Deviation
IQR	Interquartile Range
GLMMs	Generalized Linear Mixed-Effects Models
ORs	Odds Ratios
95% CIs	95% Confidence Intervals
MRs	Mean Ratios
min/week	Minutes per Week
